# The Effect of *Prosopis farcta* and Its Bioactive Luteolin on the Hippocampus of Mice after Induced Ischemia Reperfusion

**DOI:** 10.1155/2022/8157948

**Published:** 2022-01-24

**Authors:** Shahram Mohammadpour, Fereshteh Ghiasyzadeh, Marzieh Darvishi, Elahe Karimi, Hori Ghaneialvar, Rafieh Alizadeh, Ardeshir Moayeri, Naser Abbasi

**Affiliations:** ^1^Department of Anatomy, Medical School, Ilam University of Medical Sciences, Ilam, Iran; ^2^Biotechnology and Medicinal Plants Research Center, Ilam University of Medical Sciences, Ilam, Iran; ^3^ENT and Head and Neck Research Center and Department, The Five Senses Health Institute, School of Medicine, Iran University of Medical Sciences, Tehran, Iran; ^4^Department of Pharmacology, Faculty of Medicine, Ilam University of Medical Sciences, Ilam, Iran

## Abstract

**Background:**

Ischemia plays an important role in increasing damage to the nervous system. This study aimed to evaluate the effect of *Prosopis farcta* (PFE) and its bioactive luteolin (Lu) and forced swimming exercise on the hippocampus of mice after induced ischemia reperfusion.

**Methods:**

The bioactive component of PFE (Lu) was identified by HPLC. Fifty-six male mice were divided into different groups. Ischemia was induced by ligation of the common carotid artery. After mice training (swimming exercise, 8 weeks) and consuming PFE and Lu, the mice's memory ability was evaluated in the shuttle box. Histological examination was performed by Nissel staining and immunohistochemistry.

**Results:**

Results showed that the ischemic mice exercised and treated with PFE and Lu had higher step-through latency (STL) compared with the nonexercised mice, and this was confirmed with time spent in the dark compartment (TDC). The number of dark cells in the ischemic group exercising and receiving PFE and Lu decreased compared to that of the other groups in the hippocampus. DCX protein expression was increased in nonexercised groups compared to that of the exercised groups and those treated with PFE and Lu, while NeuN decreased.

**Conclusions:**

Forced swimming exercise following ischemia, as well as consumption of PFE and Lu, has reduced cell death and increased neurogenesis in the hippocampus and thus may help improve memory in ischemia.

## 1. Introduction

Stroke is one of the leading causes of death and the most important risk factor in the world [1]. Cerebral ischemia is a decrease in the number of brain metabolites due to a decrease in blood flow, which leads to a decrease in oxygen storage, resulting in brain tissue death or stroke [2]. During cerebral ischemia, followed by a decrease in oxygen uptake and free radicals, ATP decreases, resulting in increased free radicals and lactate, leading to acidosis and cell death. In the process of ischemic reperfusion, reactive oxygen species (ROS) and reactants such as peroxide hydrogen and radical hydroxide (OH¯) are produced, which attack the target tissue and severely damage it [3]. The hippocampus is an important brain structure associated with memory and learning that is highly vulnerable to ischemia. This area of the brain is supplied with blood by the anterior choroidal artery. This artery is one of the first areas of the brain to be damaged in brain diseases such as Alzheimer's, Huntington's, epilepsy, stroke, ischemia, and especially brain trauma [4]. Neurogenesis of the hippocampus is a continuous process, the process by which cells multiply in the subgranular zone (SGZ) and mature into granule cells and eventually become the neural network within the hippocampus [5]. Doublecortin protein (DCX) is one of the markers of neurogenesis that plays an important role in neuronal development in this area [6]. Neurons continue to express DCX for about 2 to 3 weeks, and then these cells begin to express NeuN (neuronal nuclei) [7]. NeuN (a marker for mature neurons) is a specific nuclear protein that is detected in immunohistochemical studies in mature neurons [8].

Animal studies have shown the beneficial effects of exercise on cerebral ischemia, including increased survival, reduced neurological defects, improved blood-brain barrier, and integrated blood vessels [9].

Some medicinal plants with multiple components have been shown to improve the injury caused by ischemia reperfusion [10]. Therefore, evaluation of the pharmacodynamic mechanisms of these compounds in the treatment of ischemia can be valuable. The plant of *Prosopis farcta L.* (Leguminosae) is widespread in different dry and semidry regions worldwide including Africa, Australia, America, and Asia [11]. Some of the phytochemical components in this plant include quercetin, tryptamine, apigenin, 5-hydroxytryptamine, L-arabinose, lectin, and luteolin [12]. Luteolin, a bioflavonoid (2-(3, 4-dihydroxy phenyl)-5, 7- dihydroxy-4-chromanone), is known to be present in many types of plants and possesses diverse biological properties including antioxidant, anti-inflammatory, and anticancer activities [13]. It is also proven to attenuate anxiety [14] and ameliorate amnesia [15]. It has been demonstrated that the administration of luteolin prevented ischemia-reperfusion injury through a rebalancing of pro-oxidant/antioxidant status [16]. So, an investigation of the potential neuroprotective role of luteolin in ischemia reperfusion and the underlying mechanisms can be considered. Also, according to many studies on the reduction of damage to the hippocampus after the induction of ischemia reperfusion, the effect of exercise neurogenesis on the hippocampus before ischemia has been proven [9], but so far the effect of force swimming exercise on comprehensive research has not been performed after the induction of ischemia reperfusion. In this study, the effect of force swimming exercise in comparison with PFE and its bioactive component Lu on the male mouse hippocampus after induction of ischemia reperfusion was investigated.

## 2. Materials and Methods

### 2.1. Plant Material and Extraction

In the fruiting season, the aerial parts of *Prosopis farcta* were collected from Ilam Province, Iran (November 2018). The plant specimen voucher number was deposited in Birjand University, Iran with herbarium code 74/1525. The samples (50 grams) were powdered after drying and extracted by the Soxhlet extraction method in a water-methanol solvent. After extraction, the solvents were evaporated by a rotary (IKA HB 10, Germany), and the yield of extraction was 7.84%. Then, it was lyophilized and stored at −20°C. The samples were dissolved in methanol and filtered through a 0.22 *μ*m syringe filter [17].

### 2.2. High-Performance Liquid Chromatography Analysis of Luteolin in PFE

According to the reported procedure, the high-performance liquid chromatography (HPLC) method was conducted [18]. A reversed-phase HPLC with Knauer liquid chromatography (Smartline; Knauer, Berlin, Germany) equipped with a UV detector (WellChrom, K-2600; Knauer) and a C18 column (Nucleosil H. P.; 25 cm–0.46 cm internal diameter, pore size 0.1 mm; Knauer) was developed and validated for the determination of luteolin (Lu). Column temperature, mobile phase (methanol (A) and 0.1% formic acid in water (B)) flow rate, injection volume, and detection wavelength were all set at 25°C, 1 mL/min, 5 *μ*L, and 348 nm, respectively. A luteolin standard solution (dissolved in methanol) was run in the same condition. 250 mg of plant sample were dissolved in 10 ml methanol, sonicated for 15 min, filtered through a 0.22 *μ*m syringe filter, and further diluted to 5 mg/ml. The obtained peaks from the *Prosopis farcta* extract (PFE) were compared with standard Lu (0.1, 0.2, 0.4, 0.8, 1.6, 3.2, 6.4, 12.5, 25, and 50 *μ*g/ml).

### 2.3. Animal Experiments

This study was performed on 56 male mice with a weight of 25 to 30 grams. The animals were easily accessible to standard water and pellet diet and were kept in standard plastic cages (12 h of darkness and 12 h of light) at an optimum temperature of 24 ± 1°C. It was given 10 days before the study time to acclimatize them to the laboratory environment. The ethical approval for this study was obtained from the Animal Care and Ethics Committee (ACEC) of the Ilam University of Medical Sciences (IR.MEDILAM.REC.1395.65). All rats were randomly divided into 8 equal groups (*n* = 7) as follows: (1) the sham group that exercised: a group in which ischemic induction had not been performed, and they had been given force swimming exercise for 2 weeks; (2) the sham group that did not exercise: they underwent surgery but did not induce ischemia; (3) the sham group treated with PFE; (4) the sham group treated with Lu; (5) the ischemia group that exercised: a group that was given force swimming exercise after the induction of ischemia; (6) the ischemic group that did not exercised: a group that underwent surgery, and the joint carotid arteries were temporarily closed, (7) the ischemic group treated with PFE; and (8) the ischemic group treated with Lu.

### 2.4. Ethics Declaration

Local/Institutional: Ilam, Iran.

Name: Ilam University of Medical Sciences (IR.MEDILAM.REC.1395.65).

National Academies Press (NAP) Guide for the Care and Use of Laboratory Animals.

### 2.5. Calculation of Median Lethal Dose (LD50) in Rats

The rate of mortality and symptoms of toxicity for each group of rats were observed after 24 hours. Then, for the calculation of LD50, expired animals were counted by the Karber method [19]. For this purpose, different doses of the PFE (50, 100, 200, 300, 400, 500, 600, and 700 mg/kg) and luteolin (10, 20, 30, 60, 90, 120, 150, and 180 mg/kg) dissolved in normal saline were gavaged in the groups. Therefore, PFE (150 mg/kg) and Lu (30 mg/kg) were selected.

### 2.6. Two-Vessels Occlusion Mouse Model of Cerebral Ischemia Reperfusion

To create an ischemic model, the animal was first anesthetized using a combination of ketamine (100 mg/kg) and xylazine (5 mg/kg) [20]. Ischemia was then performed by closing the arteries of the common carotid artery. The animal's head was fixed on a surgical board, then a vertical incision was made in the centerline of the neck with scissors, and after the thyroid gland was removed, the omohyoid muscle was exposed. After opening the carotid sheath, the common carotid artery carefully separates from the vagus nerve and the internal jugular vein and is blocked by microbleeding clamps arteries on both sides for 20 minutes. After this time, the clamps were opened to re-establish blood flow, and the animals were monitored until they regained consciousness and stabilized [21]. In the sham model, after anesthetizing the mice and cutting in the anterior neck and removing the thyroid gland and related muscles, the articular carotid artery was exposed and the incision site was closed without ischemic induction.

### 2.7. Forced Swimming Exercise

The animals were immersed in a round 100 cm in diameter and 50 cm high plastic container with a water depth of 35 cm and a temperature of 25 ± 2°C [22, 23]. In the first week, to reduce the stress of swimming and adaptation to the training environment, 20 minutes of swimming exercise per day was given 5 times a week, and from the second week, 25 minutes of exercise, then 5 minutes of rest, and then another 25 minutes of exercise were given for 8 weeks. After the exercise, the mice were blow-dried [24]. Mice were excluded from swimming when they were unable to stay afloat after repeated attempts or remained underwater for ten seconds [25].

### 2.8. Shuttle Box Test

Passive avoidance learning was used to measure the ability to learn and function memory by using the shuttle box. The device consists of two separate chambers (20 × 30 cm) and a height of 20 cm, which are connected by a guillotine valve. One of the rooms is lit by a 40-watt ceiling lamp, and the other is a dark room. At the bottom of both sections, there are metal rods that are one millimeter in diameter and one centimeter apart and can be used with a stimulator. To learn, the mouse was placed in a bright chamber, and after 30 seconds of the mouse getting used to the chamber, the door between the two rooms was opened and the mouse was allowed to enter the darkroom, where an electric shock with an intensity of 0.25 mA– 20 watts entered the animal's feet for 2 seconds. The time elapsed before entering the darkroom was recorded. The animal came out of the dark a few seconds later. The delay in entering the step-through latency (STL) dark section was then measured and compared between groups. The test ended when the mouse avoided entering the dark part for 300 seconds [26].

### 2.9. Histological Examinations

#### 2.9.1. Tissue Processing

The animals were deeply anesthetized i.p. with a high dose of ketamine (150 mg/kg) with a midline abdominal incision in the skin of the anterior abdominal wall. Following the washing of the arteries and the lightening of the color of the output solution, the 4% paraformaldehyde fixative solution dissolved in the phosphate solution of one-tenth of a molar (pH = 7.4) was passed through gravity using gravity for 10 to 15 minutes. After the perfusion of the animal's head was separated, the brain was carefully removed and placed in a secondary fixative solution [27].

#### 2.9.2. Nissl Staining

After fixation and preparation, sections with a thickness of 7 *μ*m and a distance of 2.3 to 5 mm from the posterior of the bregma were prepared using a rotary microtome placed on the gelatinized slides and stained using the Nissl method. The samples were analyzed using an optical microscope with a 400*x* magnification. Only neurons with a clear nucleus and nucleolus were considered as viable and healthy cells. For each sample, ten photomicrographs with a minimum distance of 40 *μ*m were randomly selected. The pyramidal cells in the CA1 region were counted by Image Tools 2 software, and the mean counts were determined [28, 29].

### 2.10. Immunohistochemistry

Tissue sections (7 *μ*m) immersed in paraffin, fixed by formalin, were deparaffinized with xylene, rehydrated with ethanol, and boiled at 96–98°C in a bain-marie in citrate buffer (0.01 M and pH 6.0). With hydrogen peroxide (3%), endogenous reactive oxygen species were suppressed (40 min). Samples were blocked with a goat (DCX, NeuN) for 1h. Immunohistochemistry was carried out and counterstaining was performed with hematoxylin. From the same specimens, the negative control section was processed, with irrelevant IgG of the same species (replacing the primary antibody) and concentration as the primary antibody [30].

### 2.11. Statistical Analysis

All results are reported as a mean ± SD. The Kolmogorov–Smirnov test was used to verify the normality of the distribution. A one-way analysis of variance (ANOVA) test was used to compare the differences between the groups. The level of significance was set at *P* < 0.05. All data were analyzed by the SPSS software (SPSS for Windows; SPSS Inc., Chicago, IL, USA; Version 16.0).

## 3. Results

### 3.1. Extraction and Identification

**Figure 1:**
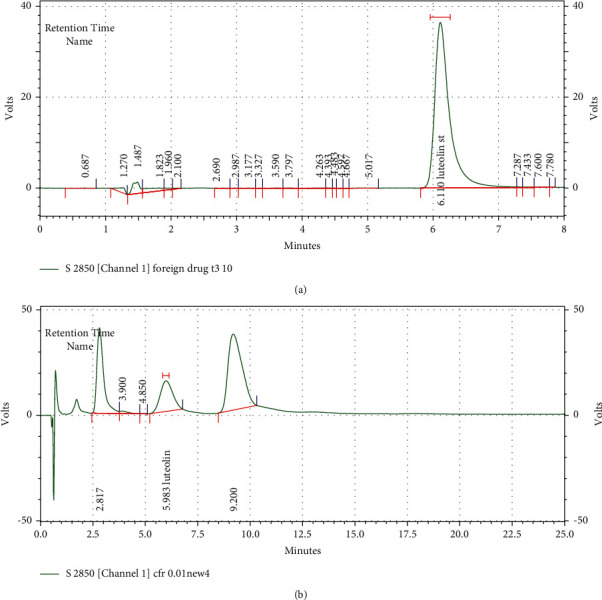
Standard luteolin (Lu) HPLC chromatogram (a) and *Prosopis farcta* extract (PFE) (b). HPLC conditions were the same for both of them.

The HPLC chromatogram of standard Lu was obtained with a retention time of 6.110 min (348 nm). The chromatogram of the methanolic PFE was similar to that of Lu in 5.983 min, in the same conditions (Figures 1(a) and 1(b)). Lu was found to be in methanol fraction of PFE (1.52 mg/g of Lu, *R*^2^ = 0.9995, *y* = 156478*x* − 3967.01).

### 3.2. Determination of Median Lethal Dose

The median lethal dose (LD_50_) of PFE and Lu was 565 and 180 mg/kg, respectively (within 72 hours, i.p.). The mortality rate increased in a dose-dependent manner. No mortality was reported in animals administered orally with PFE (150 mg/kg) and Lu (30 mg/kg).

### 3.3. Comparison of Step-Through Latency (STL)

**Figure 2:**
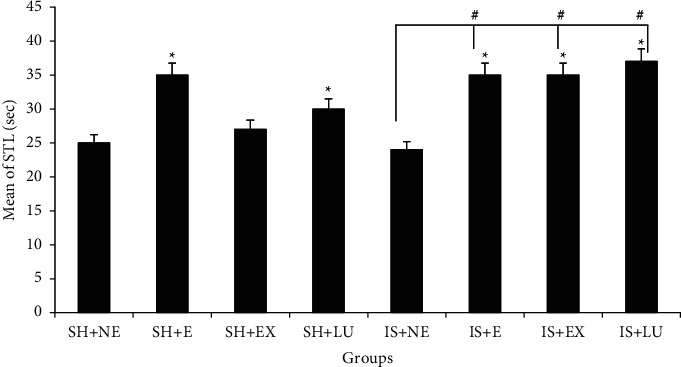
Comparison of delay time in entering the dark part (STL) of the shuttle box after receiving the shock. (SH + NE) sham not exercised, (SH + E) exercised sham, (SH + EX) sham-treated with *Prosopis farcta* extract, (SH + LU) sham-treated with luteolin, (IS + NE) not exercised ischemia, (IS + E) exercised ischemia, (IS + EX) ischemia treated with *Prosopis farcta* extract, (IS + LU) ischemia treated with luteolin. ^*∗*^*P* < 0.01 vs SH + NE, # vs (IS + NE).

A comparison of the values obtained from this behavioral test between sham and ischemia groups showed that there is a significant difference between forced swimming exercise and treated groups with PFE and Lu (Figure 2). Results showed that those who were forced to do swimming exercise had a higher STL than those who did not exercise (*P* < 0.01).

### 3.4. Comparison of Time Spent in the Dark Compartment (TDC)

**Figure 3:**
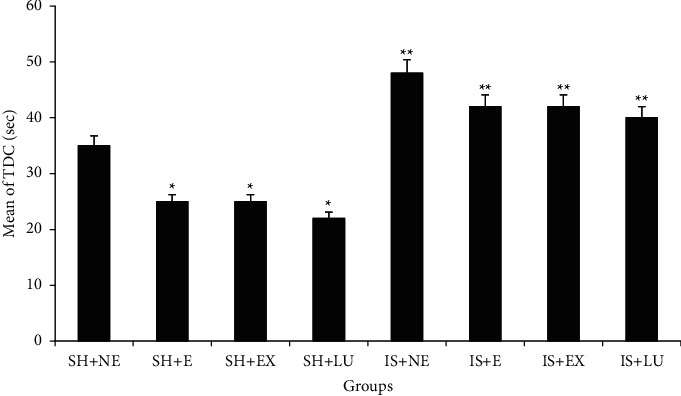
Passive avoidance memory test; average total time spent in the dark chamber (seconds) in the reminder phase in different groups; (SH + NE) sham not exercised, (SH + E) exercised sham, (SH + EX) sham-treated with *Prosopis farcta* extract, (SH + LU) sham-treated with luteolin, (IS + NE) not exercised ischemia, (IS + E) exercised ischemia, (IS + EX) ischemia treated with *Prosopis farcta* extract, (IS + LU) ischemia treated with luteolin. ^*∗*^*P* < 0.01 vs SH + NE.

Comparisons between exercised and treated with PFE and Lu groups and those who did not exercise and did not treat with PFE and Lu showed that those who did not exercise had a higher TDC index (*P* < 0.01) (Figure 3). A significant difference was observed in the comparison between the nonexercised sham and ischemic groups (*P* < 0.01).

### 3.5. Histological Results

#### 3.5.1. Chrysalis Violet Coloring

Cell counting in three hippocampus areas (CA_1_, DG, and CA_2, 3, 4_) was performed by microscope. The number of damaged cells (dark cells) in these areas was compared. In Niesel staining photographs, necrotic cells were characterized by irregularly shrunk nuclei and intense and uniform staining with chrysalis violet. Results showed that the largest number of dark cells in the ischemic group did not exercise, and the lowest number in the sham group did not exercise.

**Figure 4:**
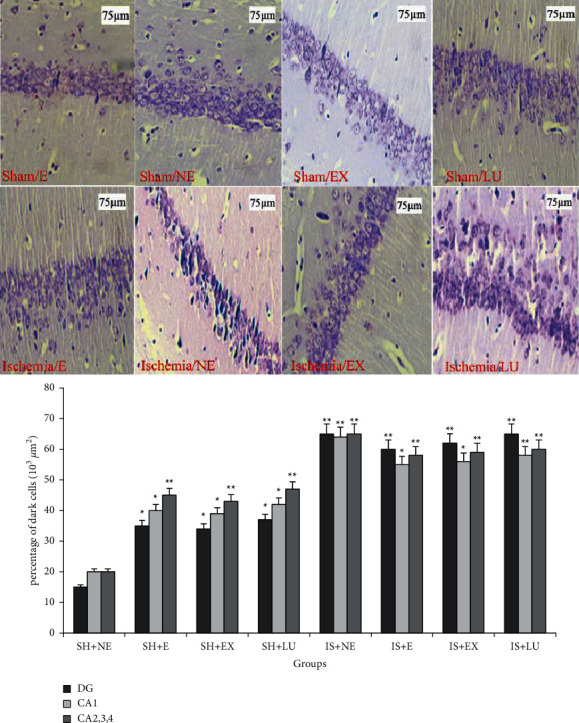
Comparison of the mean number of dark cells in hippocampus regions (CA_1_, DG, CA_2,3,4_) in the studied groups; (SH + NE) sham not exercised, (SH + E) exercised sham, (SH + EX) sham-treated with *Prosopis farcta* extract, (SH + LU) sham-treated with luteolin, (IS + NE) not exercised ischemia, (IS + E) exercised ischemia, (IS + EX) ischemia treated with *Prosopis farcta* extract, (IS + LU) ischemia treated with luteolin. ^*∗*^*P* < 0.01 vs SH + NE, ^*∗∗*^*P* < 0.001 vs SH + NE.

The average number of dark cells in all parts of the hippocampus in ischemic groups has increased compared to sham groups (both in the exercise and treated with PFE and Lu groups and in the nonexercise group and the nontreated group) (*P* < 0.01). Also, comparisons between different areas of the hippocampus showed that the lowest rate of pyknotic cells was in the DG region (Figure 4).

### 3.6. Immunohistochemistry

**Figure 5:**
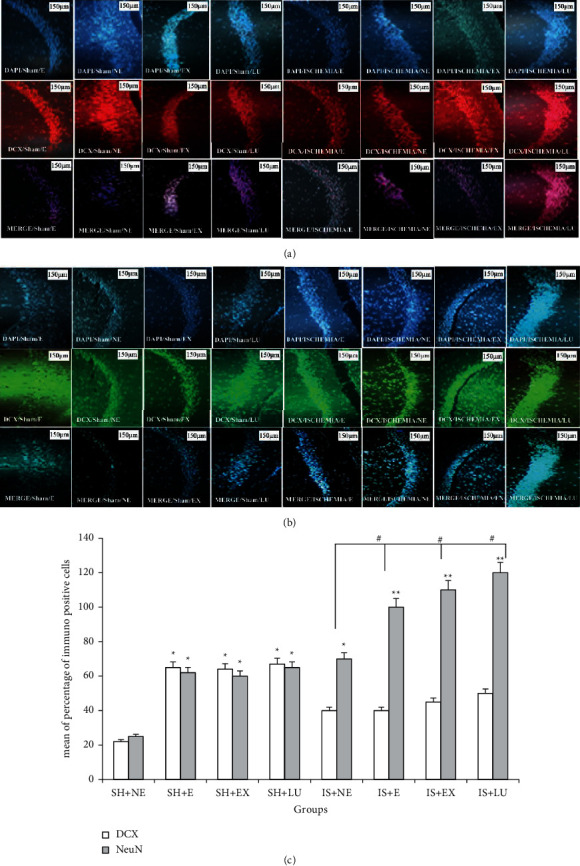
Immunofluorescence staining of neuron marker. (a) (DCX), (b) NeuN, and (c) mean of the percentage of immunopositive cells. Immunofluorescence assay was performed for DAPI (A), DCX, NeuN (B), and MERGE (C) in CA1 hippocampal brain incisions in different groups on day 57 (a, b); (SH + NE) sham not exercised, (SH + E) exercised sham, (SH + EX) sham-treated with *Prosopis farcta* extract, (SH + LU) sham-treated with luteolin, (IS + NE) not exercised ischemia, (IS + E) exercised ischemia, (IS + EX) ischemia treated with *Prosopis farcta* extract, and (IS + LU) ischemia treated with luteolin. ^∗^*P* < 0.01 vs SH + NE, ^∗∗^*P* < 0.001 vs SH + NE.

The highest expression of DCX in the CA_1_ region was in the sham group. Also, in ischemic groups, the rate of expression of this marker was not higher than that in exercised and treated with PFE and Lu groups. NeuN's highest expression in the CA_1_ region was in the ischemic group treated with Lu, and the lowest expression was in the nonexercised sham group (Figure 5).

The expression of DCX protein in sham groups in the exercised and treated with PFE and Lu groups increased compared to that of the nonexercise group, and since this marker represents neurogenesis, it seems that exercise has increased neurogenesis and cell division, but in ischemia, the observations were reversed.

It has been shown that the NeuN marker in nonexercised sham groups was more than that in the exercised and treated with PFE and Lu groups, which could be due to increased neurogenesis followed by cell maturation. However, in the ischemic group, the expression of NeuN protein was higher in the exercise and treated with PFE and Lu groups than in the nonexercise group.

## 4. Discussion

To study the therapeutic effects of forced swimming exercise and PFE and its bioactive Lu on the hippocampus of mice after induced ischemia reperfusion was investigated. The findings of the present study showed that 8 weeks of forced swimming exercise and PFE and Lu improved passive avoidance learning in ischemic mice.

In this study, the HPLC chromatogram of luteolin was obtained with a retention time of 6.110 and 5.983 min for Lu and PFE, respectively, at a wavelength of 348 nm as a standard broad peak. It has previously been reported that one of the active components of PFE is Lu [31]. According to quantitative analysis, luteolin was found to be predominant in the ethanol fraction (1.52 mg/g) of PFE. In a previous study, the amount of luteolin in PFE was reported to be 2.64 mg/g [32].

It has been demonstrated that damage to the hippocampus following ischemia may lead to behavioral defects, especially memory and learning disabilities [33]. It has been shown that exercise improves passive avoidance learning in hippocampus ischemic mice [34]. Another study revealed the effect of exercise on cognitive function in rodents, which showed an improvement in learning in exercised animals [35]. Our study found that temporary induction of ischemia also caused extensive neuronal damage in the hippocampus as well as learning and memory impairments. Also, the findings of the present study showed that 8 weeks of forced swimming exercise and PFE and its bioactive Lu improved memory impairment in ischemic mice.

Comparisons between exercised and nonexercised mice in this study showed that exercise had a higher STL. Also, the results showed that consumption of PFE and its bioactive Lu increased STL. Moreover, comparisons between exercised and nonexercised mice in the present study showed that the nonexercised had a higher TDC than the exercised. Also, the results showed that consumption of PFE and its bioactive Lu decreased TDC.

Although it has been shown that intense (2 weeks) and long-term (8 weeks) physical activity with stress does not affect spatial memory and learning [36]. Nevertheless, according to our results, exercise in normal conditions by increasing neurons and reducing apoptosis has improved passive avoidance learning [37].

Although so far no study has been found on the role of PFE in ischemia-induced learning, nevertheless, in the plant family (*Fabaceae*) such as *Erythrophleum ivorense* [38] and *Erythrina velutina* [39] extracts have been shown to improve dose-dependent in spatial and functional learning in rats by the shuttle box technique.

Besides, according to our results, Lu has shown neuroprotective function against ischemia reperfusion [40] and has also improved learning in those with Alzheimer's disorders and defects in the hippocampus [41].

It has previously been shown that aerobic exercise in rats increases spatial learning and neuronal density of the hippocampus without altering apoptosis and improves short-term memory [42]. However, the present study showed that the number of pyknotic cells (dark cells) in the forced swimming control groups was more than in those that did not exercise. In confirmation of our results, it has been shown that increased oxidative stress during strenuous exercise has impaired mitochondrial electron transfer and resulted in cell death [43].

The effects of PFE on the number of dark cells in the dose used in this project showed its protective effects on nerve cells. However, this plant extract had anticancer effects via mitochondrial alterations, as exemplified by increased ROS levels in high concentrations [44] and hepatoprotective activity in low doses [45]. Our results showed that luteolin increased cellular protection. Although luteolin has been shown to increase cell death in PC12 cells [46] and primary neurons [47] at high concentrations, it has cell protective effects on human neuroblastoma [48] and amyloid *β* protein in cortical neurons cells [49] at lower concentrations.

The results of DCX immunohistochemical staining in the CA_1_ area of the hippocampus showed that the number of DCX cells was increased in the forced swimming exercise sham group. Thus, it has been suggested that swimming activity increases neurogenesis in the CA_1_ region. In agreement with our studies, it has been shown that exercise increases the number of new cells in the hippocampus and improves brain function in rats [50]. Also, physiological studies have shown that physical activity increases the electrical activity of the hippocampus, which can be caused by changes in physical activity and neurotransmitters [51]. Our results demonstrated that in the nonexercise ischemia group, the number of DCX was higher. The cause of this condition could likely be due to increased brain damage and cell proliferation in the hippocampus, around the affected area of the cortex, and in the ventricular area of the lateral ventricles [52]. In the following, it has been shown that this cell proliferation was more towards the production of neuroglia cells, and that the beneficial effects of exercise on cognition and mood were mediated by an increase in new cells in the hippocampus [53]. In this study, it was shown that although the level of DCX in the exercised ischemia group decreased due to the antioxidant induction, this cell increase in the nonexercise group went more towards glial cell production, and therefore, in the evaluation of the NeuN marker (shows mature neurons) decreased in the nonexercise group, as most cells went to produce astrocytes and neuroglia cells.

Astrocytes and Betz neurons in CA_1_ regions have been shown to undergo apoptosis as an increase in caspase 12 following endoplasmic reticulum stress-dependent postischemic mechanisms [54]. Another study found that stress reduces the expression of neurogenesis-related genes, thereby reducing cell proliferation in the brain [55]. The results of NeuN immunohistochemical staining in the CA_1_ area of the hippocampus showed that the number of NeuN cells increased in the exercised sham group. This suggests that forced swimming exercise increases the differentiation of endogenous stem cells in the CA_1_ region.

The results of this study showed that in the CA_1_ region, the expression of DCX cells in the ischemic groups that received PFE and Lu decreased compared to that in the control group, while the number of NeuN cells increased significantly.

Although a study of neurogenesis in ischemic reperfusion has not yet been found for the PFE and Lu, it has been shown that flavonoids such as hepta methoxy flavone [56], 3, 5, 6, 7, 8, 30, 40-heptamethoxyflavone [57], and baicalin [58] in the ischemic state can stimulate and enhance neurogenesis in the hippocampus in animals.

## 5. Conclusions

The present study concluded that forced swimming exercise and consumption of PFE and Lu reduced cell death and increased neurogenesis in the ischemia-reperfusion injury hippocampus, thereby improving memory function.

## Data Availability

The datasets generated during and/or analyzed during the current study are avaiable.
